# Early Diagnostic Markers in Crisponi Syndrome: Two Cases and Review

**DOI:** 10.3390/jcm14217757

**Published:** 2025-11-01

**Authors:** Lorenzo Perilli, Kamil Dzwilewski, Marta Pietruszka, Pasquale Striano, Giuseppe Capovilla, Maria Mazurkiewicz-Bełdzinska

**Affiliations:** 1 Clinical Pediatrics, Department of Molecular Medicine and Development, Azienda Ospedaliero-Universitaria Senese, University of Siena, 53100 Siena, Italy; 2 Department of Neurosciences, Rehabilitation, Ophthalmology, Genetics, Maternal and Child Health, University of Genoa, 16126 Genoa, Italy; 3 Department of Developmental Neurology, Medical University of Gdańsk, ul. Dębinki 7, 80-952 Gdansk, Poland; 4 Paediatric Neurology and Muscular Disease Unit, IRCCS Instituto “G. Gaslini”, 16147 Genova, Italy; 5 Poliambulanza Foundation Hospital Institute, 25124 Brescia, Italy; giuseppe.capovilla@poliambulanza.it

**Keywords:** prenatal diagnosis, Crisponi syndrome, Neurogenetics, cold-induced sweating syndrome

## Abstract

**Background:** Crisponi/cold-induced sweating syndrome (CS/CISS) is a rare autosomal recessive disorder characterized by severe neonatal manifestations including paroxysmal muscle contractions, tendency for hyperthermia, and feeding and swallowing difficulties with high neonatal mortality. Pathogenic variants in the Cytokine Receptor-Like Factor 1 (*CRLF1*) gene have been associated with CS/CISS. These variants result in a loss of function of the encoded protein, which disrupts the formation of a functional heterodimer with Cardiotrophin-Like Cytokine Factor 1 (CLCF1). This complex is essential for the development of autonomic and sensory nervous systems, as well as for bone remodeling. We report two patients affected by CS harboring pathogenic variants in the *CRLF1* gene. **Methods—case reports:** The first patient was diagnosed postnatally, presenting with non-epileptic paroxysmal events characterized by opisthotonus and orofacial contractions. He survived beyond infancy, later developing scoliosis and persistent episodes of hyperthermia. In the second patient, a prenatal ultrasound at 20 weeks of gestation revealed bilateral camptodactyly, also referred to as the ‘horn’s sign’, raising early suspicion of CS. The diagnosis was subsequently confirmed both clinically and genetically. After birth, the infant developed severe dysphagia, apnea, and paroxysmal events not associated with epileptiform activity on EEG. Sanger sequencing identified a homozygous c.708_709delinsT frameshift variant in the *CRLF1* gene. The patient died at 30 days of age due to respiratory failure. **Results and conclusions:** With this manuscript, we aim to further delineate the phenotypic spectrum of this rare condition and propose the ‘horn’s sign’ as a targeted prenatal marker for early diagnosis in populations with known founder mutations or familial risk factors.

## 1. Introduction

CS/CISS is a rare autosomal recessive genetic condition with high neonatal lethality, originally reported in 1996 among a group of 12 Sardinian families [1].

This rare condition is evident from birth, presenting with contractions of the oropharyngeal muscles, abundant salivation, and characteristic facial expression. Importantly, during these episodes there is a high risk of suffocation, leading to apnea of variable duration and generalized, moderate hypertonia with opisthotonus. These episodes are triggered by crying, pain or light stimuli and never occur during sleep or in quiet states. They disappear with simple tranquillization and administration of oxygen. CS/CISS patients experience serious hyperthermal peaks above 42 °C, which are occasionally followed by generalized convulsions. Newborns with CS also experience impaired swallowing, which leads to feeding difficulties, gastroesophageal reflux, and respiratory complications [1,2,3,4].

The clinical course is generally fatal, leading to death in the first days or weeks after birth because of severe paroxysmal attacks or hyperthermic crises [5].

Distinct dysmorphic facial features include a round face with full or puffy cheeks, low-set ears, a depressed nasal bridge, anteverted nares, a long and smooth philtrum, a high-arched palate, micrognathia, and a characteristic ‘carp-like’ mouth with a curled upper lip. Dental anomalies such as delayed eruption, hypodontia, malocclusion, odontogenic cysts and bruxism have also been described [1,2,3,4].

The most distinctive and pathognomonic marker of CS is the ‘horn’s sign’, which is the presence of hands’ bilateral camptodactyly [6].

We report on 2 new patients who manifested symptoms of CS/CISS at birth, with pathogenic variants in CRLF1 gene (Table 1). Through this manuscript, we seek to expand the phenotypic characterization of this rare condition and propose the ‘horn’s sign’ as the most distinctive and clinically actionable early marker of CS, as it may guide early detection and clinical management, particularly in populations with known founder mutations or familial risk factor.

## 2. Materials and Methods

### 2.1. Literature Search and Selection Criteria

This narrative review was designed to summarize current clinical and molecular knowledge on Crisponi/cold-induced sweating syndrome (CS/CISS). Relevant literature was retrieved through iterative searches in PubMed and Scopus, using combinations of the terms “Crisponi syndrome” cold-induced sweating” and “prenatal diagnosis”, without time restrictions and limited to English peer-reviewed publications. Other databases such as Embase and Web of Science were not included. Additional references were identified by screening bibliographies of selected articles. Case reports, case series, and reviews providing clinical or molecular data were included.

### 2.2. Genetic Analysis 

Genomic DNA was extracted from peripheral blood samples of the patients and their parents. All nine coding exons and flanking intronic regions of the CRLF1 gene were amplified and analyzed using Sanger sequencing. Variant interpretation followed the American College of Medical Genetics and Genomics (ACMG) guidelines, with classification supported by ClinVar and population databases (e.g., gnomAD). Identified variants were confirmed in parents to determine zygosity and inheritance pattern. Informed consent for publishing clinical data and videos was obtained from parents of the described patients.

## 3. Case Descriptions

### 3.1. Patient #1

The patient of Italian origin was born at term, with an APGAR score of 10 at the first minute of life, after an uncomplicated delivery. The child exhibited round face with full cheeks, a poorly developed and depressed nasal bridge, anteverted nares, and a long philtrum. Moreover, bilateral camptodactyly was present, with fisted hands and abnormal arrangement of toes. A few hours after birth, the newborn experienced paroxysmal episodes characterized by increased muscle tone, hyperreflexia, opisthotonus, spasms and generalized tremors. Muscle contractions, particularly involving the facial and oropharyngeal muscles, were triggered by sudden external stimuli such as crying or pain. These episodes resolved once the stimuli ceased, after rest and with oxygen administration. While being handled the child tended to grimace and startle, followed by retraction of the head and flexion of the arms. The patient subsequently developed severe dyspnea, alternating with apnea episodes, as well as trismus and abundant sialorrhea. Further, because of severe dysphagia, he required the placement of a nasogastric tube.

Serum infection markers and metabolic tests yielded negative results, head CT and brain MRI were unremarkable. Electrocardiogram, electromyography, and muscle biopsy resulted normal. Due to suspect of Crisponi syndrome, Sanger sequencing detected two heterozygous *CRLF1* variants—c.226T>G (p.W76G) in exon 2 and C.676-677insA (p.T226NfsX104) in exon 4, interpreted as pathogenic.

In the following years the patient continued to exhibit severe hyperthermia characterized by heat intolerance and paradoxical sweating, unresponsive to antipyretic drugs. Only physical measures—such as immersion in cold water or frequent changes of clothing—proved effective. Furthermore, the clinical history was complicated by both gastroenterological and orthopedic complications. The child presented with feeding difficulties, including gastroesophageal reflux, which was exacerbated by paroxysmal episodes of orofacial muscle contractions during crying. Additionally, an elbow malformation limited flexion, and scoliosis required major corrective surgery at the age of 10. The patient was subsequently lost to follow-up, and no further clinical information is available regarding long-term outcome.

### 3.2. Patient #2

**Figure 1:**
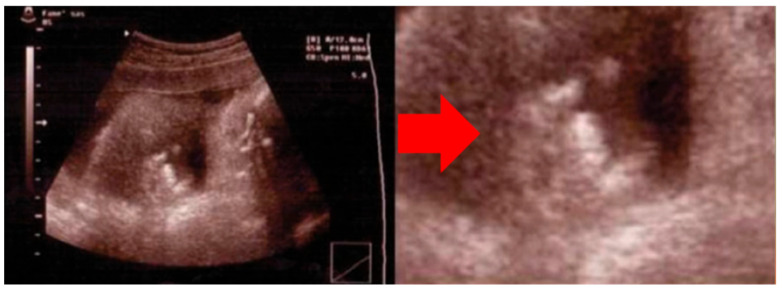
Demonstrative fetal ultrasound image showing prenatal camptodactyly (“horn’s sign”), with detail of the fetal hand indicated by a red arrow. This image is used for illustrative purposes. Reproduced and adapted from Dessì et al., J. Obstet. Gynaecol. Res. 2012;38:582–585 [6].

In this child, suspicion of CS/CSS was raised prenatally, as a routine ultrasound examination at 20 weeks of gestational age revealed bilateral camptodactyly or “horn’s sign” pathognomonic for CS/CISS (Figure 1). This evidence prompted prenatal counselling, also taking into account that the parents were first cousins, indicating parental consanguinity. The parents originated from northern Italy. The patient was admitted to the neonatal intensive care unit two hours after birth due to early-onset achalasia, severe dysphagia, and excessive salivation with foamy secretions, which required nasogastric tube placement. Additionally, the child experienced laryngospasm with episodes of dyspnea and apnea, later complicated by aspiration pneumonia. On general examination, facial dysmorphic features, trismus, and bilateral camptodactyly were observed.

**Figure 2:**
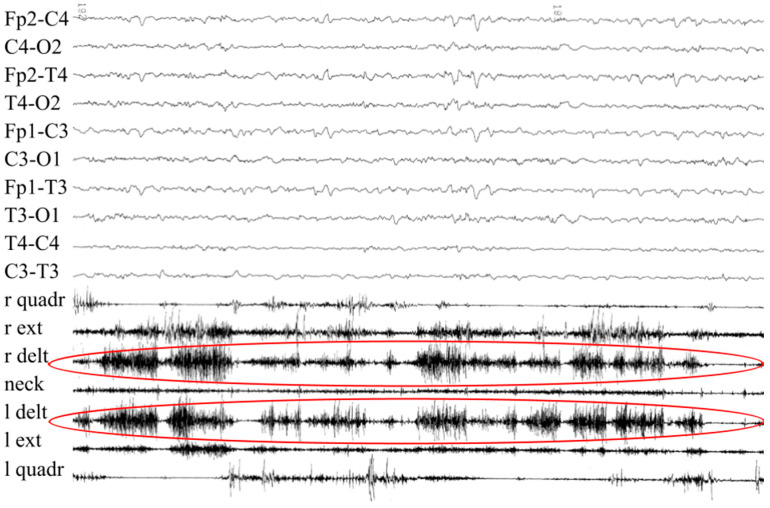
Patient’s #2 EEG performed during paroxysmal events showed no epileptiform discharges. Instead, the observed muscle contractions (red circles) were consistent with stimulus-induced hypertonia and orofacial spasms, supporting their non-epileptic nature.

During the first weeks of life, he exhibited paroxysmal episodes of hypertonia and orofacial muscle contractions, triggered by crying and resolving after calming. These events were often preceded by hyperthermia. As observed in the first patient, body temperature responded only to physical measures. Polysomnography and EEG performed during a paroxysmal apneic event confirmed a mixed (central and obstructive) apnea, while epileptiform activity was excluded (Figure 2).

Direct sequencing of the nine coding exons and flanking intronic regions of the *CRLF1* gene, using specific primers, identified a pathogenic c.708_709delinsT homozygous variant, which causes a frameshift in the second fibronectin type III domain (p.Pro238Argfs*6). Consanguinity of the parents supported the autosomal recessive inheritance pattern.

The child survived the first weeks of life with sporadic episodes of hyperthermia, severe feeding difficulties, and frequent paroxysmal events of hypertonia and orofacial muscle contractions and eventually died at 30 days of age.

## 4. Discussion

CRLF1 (OMIM 604237), mapped to chromosome 19p13.11, encodes a soluble protein that forms a stable heterodimer with CLCF1, a pleiotropic cytokine which belongs to IL-6 family. The shared high level of conservation and expression patterns with its receptor (CRLF1), prove they are crucial for proper functioning of fetal development.

CLCF1-CRLF1 complex acts as a second functional ligand of the Ciliary Neurotrophic Factor Receptor (CNTFR), activating the homonymous pathway. This signaling cascade supports neuronal development, differentiation, and survival, and has also been implicated in the functioning of the hematopoietic, skeletal, renal, immune, and respiratory systems, both in infancy and adulthood. This explains the heterogeneous clinical presentation observed in patients with CS/CISS [2,7,8,9,10].

Crisponi syndrome usually presents in neonates with hyperthermia, often exceeding 42 °C. The exact molecular mechanism underlying this symptom remains unknown. In patients older than 6 years, however, cold-induced sweating occurs when the ambient temperature falls below 20 °C. This phenomenon has been shown to result from the role of CRLF1 and CLCF1 in the cholinergic differentiation of sympathetic neurons innervating postnatal sweat glands. To date, the only available pharmacological treatments for cold-induced sweating involve the use of α2-adrenoceptor agonists such as clonidine or moxonidine, sometimes in combination with amitriptyline [5,11]

About 80% of individuals with CS/CISS present with orofacial or laryngeal spasms. Mouse studies have shown that loss of function in either *CRLF1* or *CLCF1* reduces the number of motoneurons in the facial nucleus and lumbar ventral horn, while exogenous CLCF1 rescues these cells. Since motoneurons regulate muscle contraction and relaxation, their dysfunction likely underlies these clinical manifestations [12].

More recently, a distinct voice phenotype has also been described in CS/CISS, characterized by articulation disorders and hyper-rhinophonia. These features are probably linked to laryngospasms, neck muscle hypertonia, limited jaw movements, and palatal deformities, which are frequent in this syndrome [13].

Moreover, feeding difficulties are evident from birth due to impaired orofacial muscle function. In one study, all neonates with CS required external feeding; nearly half began solid foods only after 18 months, and problems with chewing, swallowing, and sialorrhea persisted into adolescence. Notably, none of the 14 patients observed developed normal rotatory chewing [14].

Regarding the genetic basis of this disease, the *CRLF1* variants identified in our patients were already reported in the original description of Crisponi syndrome [3]. The first case carried a compound heterozygous variant (c.226T>G; c.676_677insA), originally described in a Sardinian family. Similarly to the present case, the patient reported by Dagoneau et al. survived the first year of life but later developed severe scoliosis and mild developmental delay. These variants are associated with a known founder effect in Sardinia. The second patient carried a biallelic variant previously described in Turkish families, linked to a local founder effect in a small town in Turkey [2].

Although a definitive genotype–phenotype correlation in Crisponi syndrome remains unclear, some trends have emerged. Patients with splice-site variants or whole exon deletions affecting signal peptides often exhibit a more severe neonatal presentation with early fatal outcomes. In contrast, cases with variants located in extracellular domains, such as those reported here, may demonstrate partial phenotype expression and prolonged survival. Some CS patients survive the first year of life and may show spontaneous improvement in feeding and thermoregulation. In the long-term follow-up, psychomotor delay, orthopedic complications, or cold-induced sweating associated with elevated plasma noradrenaline levels, have been reported. As for Patient #1, he survived beyond infancy but developed scoliosis and persistent autonomic dysfunction [15,16].

Among the recurrent features, camptodactyly is commonly observed in several conditions (Table 2), including Stüve–Wiedemann syndrome/Schwartz–Jampel type 2 syndrome (SWS/SJS2), neonatal tetanus (NT), congenital contractures of the limbs and face, hypotonia, and developmental delay syndrome (CLIFAHDD), Chitayat–Hall syndrome/Schaaf–Yang syndrome (CHS/SHFYNG) [15]. Camptodactyly is a congenital, bilateral condition that occurs in isolation, without other joint involvement. It manifests at birth as a muscular contracture of the third and fourth fingers of both hands, typically affecting the proximal and, more rarely, the distal interphalangeal joints. Like the other paroxysmal muscular characteristic of CS, this manifestation is triggered by external stimuli such as touch, pain, or crying, and never occurs at rest or during sleep [1,4,8].

In CS/CISS, it is reported in over 68% of patients and often requires management with ergotherapy, bracing, or plastic surgery [15]. The specific pathophysiological mechanism remains unknown to date. Importantly, this feature can be recognized from the 20th week of gestational life on routine ultrasound, allowing early detection and clinical management of CS. In the present case series, we emphasize the importance of this clinical sign, which raised prenatal diagnostic suspicion for this rare condition and corroborates previous reports in the literature [6].

“Horn’s sign” should be considered as the central criterion in targeted prenatal screening of CS, especially among the population with known founder effect or affected by any other familial risk factors. Detecting this characteristic feature in routine prenatal ultrasound could contribute to earlier diagnosis, which, although not yet allowing for curative treatment, is crucial to ensure timely clinical management. Early recognition makes it possible to plan appropriate supportive care, optimize symptom control, and better define delivery strategies.

## 5. Future Directions

The future of patients with CS depends on the development of causative treatment strategies. Beyond precision genetic engineering, therapeutic approaches may also target the immune-related properties of the affected proteins. Potential strategies include recombinant soluble cytokine receptors or small molecules that modulate cytokine signaling, with the aim of alleviating disease symptoms. In this context, early diagnosis and prompt initiation of such therapies will be critical to improving outcomes for individuals with this rare syndrome [12].

## 6. Conclusions

This case series aims to expand the clinical spectrum of CS/CISS and contributes to understanding genotype–phenotype correlations. Moreover, we highlight the importance of differentiating non-epileptic paroxysmal events from seizures, especially in at-risk populations with known founder mutations. Furthermore, we emphasize that early recognition of Crisponi syndrome is possible through awareness of its characteristic features at birth and prenatal ultrasound signs such as the “horn’s sign”. We propose it as a central criterion in targeted prenatal screening, especially in populations with known risk factors. This enables timely diagnosis, genetic counseling, and optimized neonatal care.

This research did not receive any specific grant from funding agencies in the public, commercial, or not-for-profit sectors.

## Figures and Tables

**Table 1:** Integrated clinical and molecular summary of the described patients.

	Patient 1	Patient 2
DNA variant	Compound heterozygous: C.226T>G C.676-677insA	Homozygous: C.708_709delinsT
Exon/intron	Exon 2 Exon 4	Exon 5
Amino acid change	p.Trp76Gly p.T226NfsX104	p.Pro238Argfs*6
Clinvar accession number	VCV000005708.3 VCV000005707.4	VCV000005712.1
Reported before	Yes, both variants	Yes
Evidence prompting suspicion of cs/ciss	Orofacial muscle contractions, generalized paroxysmal events, camptodactyly	Prenatal bilateral camptodactyly

**Table 2:** Comparative features of syndromes presenting with camptodactyly.

	SWS/SJS2	NT	CLIFAHDD	CHS/SHFYNG
Inheritance	AR	N/A	AD	AD
Gene	*LIFR* 5p13.1	N/A	*NALCN* 13q33.1	*MAGEL2*, 15q11.2
Clinical features	BLB, JR, DA, HTE, RD, FSD, DD, PKS	IMT, TS, RF	LD, CD, FA, DF	ID, DPD, NH, CD, DF, FSD, FP, HTE, PSW
Difference from CS/CISS	BLB in early childhood	Continuous S without trigger, No DF	Microstomia No HTE	Overlapping phenotype with CS/CISS

Abbreviation: BLB—Bowed long bones; JR—Joint restrictions; DA—Dysautonomia; HTE—Hyperthermic episodes; RD—Respiratory distress; FSD—Feeding and swallowing difficulties; DD—Dental deterioration; PKS—Progressive kyphoscoliosis; IMT—Increased muscle tone; TS—Tetanic spasms (triggered spontaneously or by sensory stimulation); S—spasms; RF—Respiratory failure; LD—Limb deformities; CD—Camptodactyly; FA—Foot abnormalities; DF—Dysmorphic features; ID—Intellectual disability; DPD—Delayed psychomotor development; NH—Neonatal hypotonia; FP—Feeding problems; PSW—Profuse sweating.

## Data Availability

The original contributions presented in this study are included in the article/Supplementary Material. Further inquiries can be directed to the corresponding authors.

## Supplementary Materials

The following supporting information can be downloaded at: https://www.mdpi.com/article/10.3390/jcm14217757/s1, Video S1: Characteristic Postnatal Clinical Signs Video Evidence.
