# Correction: Perilli et al. Early Diagnostic Markers in Crisponi Syndrome: Two Cases and Review. *J. Clin. Med.* 2025, *14*, 7757

**DOI:** 10.3390/jcm15082809

**Published:** 2026-04-08

**Authors:** Lorenzo Perilli, Kamil Dzwilewski, Marta Pietruszka, Pasquale Striano, Giuseppe Capovilla, Maria Mazurkiewicz-Bełdzinska

**Affiliations:** 1 Clinical Pediatrics, Department of Molecular Medicine and Development, Azienda Ospedaliero-Universitaria Senese, University of Siena, 53100 Siena, Italy; 2 Department of Neurosciences, Rehabilitation, Ophthalmology, Genetics, Maternal and Child Health, University of Genoa, 16126 Genoa, Italy; pstriano@unige.it; 3 Department of Developmental Neurology, Medical University of Gdańsk, ul. Dębinki 7, 80-952 Gdansk, Poland; marta.pietruszka@gumed.edu.pl (M.P.); mmazur@gumed.edu.pl (M.M.-B.); 4 Paediatric Neurology and Muscular Disease Unit, IRCCS Instituto “G. Gaslini”, 16147 Genova, Italy; 5 Poliambulanza Foundation Hospital Institute, 25124 Brescia, Italy; giuseppe.capovilla@poliambulanza.it

## Error in Figure

In the original publication [1], there was a mistake in Figure 1 and Video S1 as published. During manuscript preparation, a published fetal ultrasound image illustrating the prenatal “horn’s sign” [2] was initially included as an example. In later drafting steps, the demonstrative image was mistakenly assumed to belong to our patient, and this oversight was unfortunately not identified before publication. The corrected Figure 1 and Video S1 appear below.

**Figure 1:**
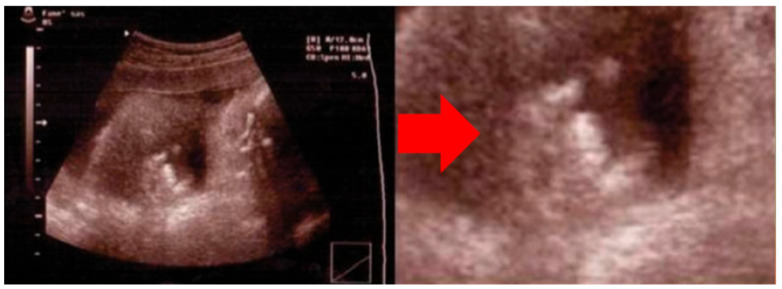
Demonstrative fetal ultrasound image showing prenatal camptodactyly (“horn’s sign”), with detail of the fetal hand indicated by a red arrow. This image is used for illustrative purposes. Reproduced and adapted from Dessì et al., J. Obstet. Gynaecol. Res. 2012;38:582–585 [2].

Video S1: Characteristic Postnatal Clinical Signs Video Evidence.

The authors state that the scientific conclusions are unaffected. This correction was approved by the Academic Editor. The original publication has also been updated.
